# Towards an Efficient CNN Inference Architecture Enabling In-Sensor Processing †

**DOI:** 10.3390/s21061955

**Published:** 2021-03-10

**Authors:** Md Jubaer Hossain Pantho, Pankaj Bhowmik, Christophe Bobda

**Affiliations:** Electrical and Computer Engineering Department, University of Florida, Gainesville, FL 32603, USA; mpantho@ufl.edu (M.J.H.P.); pankajbhowmik@ufl.edu (P.B.)

**Keywords:** CNN, embedded vision, FPGA, pixel-parallel processing

## Abstract

The astounding development of optical sensing imaging technology, coupled with the impressive improvements in machine learning algorithms, has increased our ability to understand and extract information from scenic events. In most cases, Convolution neural networks (CNNs) are largely adopted to infer knowledge due to their surprising success in automation, surveillance, and many other application domains. However, the convolution operations’ overwhelming computation demand has somewhat limited their use in remote sensing edge devices. In these platforms, real-time processing remains a challenging task due to the tight constraints on resources and power. Here, the transfer and processing of non-relevant image pixels act as a bottleneck on the entire system. It is possible to overcome this bottleneck by exploiting the high bandwidth available at the sensor interface by designing a CNN inference architecture near the sensor. This paper presents an attention-based pixel processing architecture to facilitate the CNN inference near the image sensor. We propose an efficient computation method to reduce the dynamic power by decreasing the overall computation of the convolution operations. The proposed method reduces redundancies by using a hierarchical optimization approach. The approach minimizes power consumption for convolution operations by exploiting the Spatio-temporal redundancies found in the incoming feature maps and performs computations only on selected regions based on their relevance score. The proposed design addresses problems related to the mapping of computations onto an array of processing elements (PEs) and introduces a suitable network structure for communication. The PEs are highly optimized to provide low latency and power for CNN applications. While designing the model, we exploit the concepts of biological vision systems to reduce computation and energy. We prototype the model in a Virtex UltraScale+ FPGA and implement it in Application Specific Integrated Circuit (ASIC) using the TSMC 90nm technology library. The results suggest that the proposed architecture significantly reduces dynamic power consumption and achieves high-speed up surpassing existing embedded processors’ computational capabilities.

## 1. Introduction

As convolutional neural networks (CNN) finding their way more and more into a wide range of vision-based applications, there has been a significant focus on realizing low power custom hardware accelerators to attain their services on the edge/remote devices [1,2,3,4]. However, CNNs are computationally intensive, consuming vast amounts of dynamic power and computational resources [5]. On top of that, a major challenge in deploying CNNs to the edge is the high data volume of image sensors, impacting the channel bandwidth from the sensor interface to the embedded processor [6]. With the advancement of imaging systems (i.e., cameras), the number of pixels residing in a single frame is ever increasing. For example, a video camera transmitting full HD video frames (1920 × 1080) at 30 fps will require a data bandwidth of 1492 megabits per second (Mbps). The demand is even higher for newer 4K and 8K imaging technology. Therefore, it is getting difficult to get real-time performance on embedded platforms for high-density captures.

One exciting research approach to tackle this problem is associating the processing architecture close to the image sensor and focusing resources on possible events [7]. There has been a growing interest to find methodologies to incorporate more and more computation near the vision-sensor interface. The urge can be readily sensed, as several national research agencies and funding organizations, such as DARPA, NSF, and Missile Defence Agency (MDA), have come forward, calling for novel research works in this direction [8,9]. DARPA is soliciting research proposals for fast event-based neuromorphic vision-sensors to enable intelligent sensing for DoD applications [8]. Missile Defence Agency (MDA) is seeking innovative designs for optimized bio-inspired vision sensors capable of detecting objects from high-definition images. The idea is to deploy a computation model directly in the sensor to avoid data transmission latency, minimize the bandwidth of sensor-processor communication, and alleviate the burden imposed on the backend processor.

Several approaches are discussed in the literature to bring the computational unit near the sensor and perform parallel operations [10,11,12]. The general practice is to integrate a fine grain processor with the pixel circuit, which does not deteriorate the pixel circuit’s fill-factor [13]. However, with the increase in computational complexity, the fine-grain processor’s area increases and subsequently fails the operation. Therefore, these approaches generally compute relatively simple functions near the sensor and do not offer redundancy reduction techniques that can be leveraged in vision-based CNN operations. In contrast, we aimed to integrate an inference architecture near the sensor plane.

**Figure 1:**
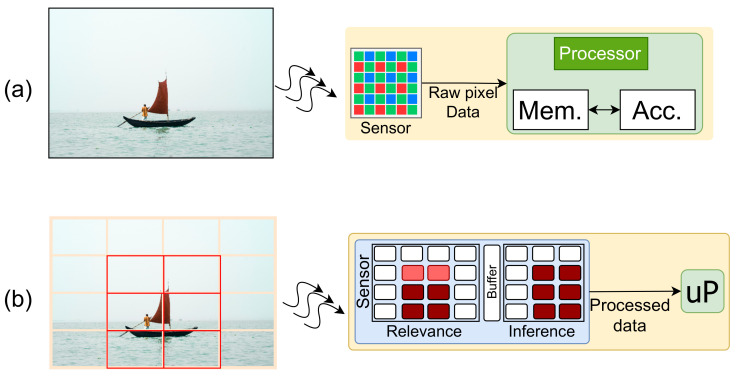
(**a**) conventional image processing system and (**b**) our proposed system; information with relevant information is processed for inference computation at the sensor.

In this paper, we introduce an event-based smart image sensor design with an integrated CNN computation layer. The proposed setup maps the sensor pixels to an array of parallel processors to facilitate CNN operations in addition to relevance detection (as shown in Figure 1b). The design aims to minimize memory, power, and computational requirements by reducing data redundancies and dynamic power consumption. The computational layer operates in a unified framework to realize a high-level application and can be re-purposed for different CNN applications. The pixel processors are specially designed to perform convolution operations and are highly optimized to hide latency and reduce external memory access. We propose a scoring based method that scores image patches based on their relevance. Here, we adopt a hierarchical optimization approach to perform computation on different regions based on their saliency score. The design assigns computational resources to different image patches based on the score. Our model emulates the biological vision system to reduce the computation cost of the architecture. The objective of this work is to support time-critical complex applications where the silicon-footprint constraint is lightly maintained.

The main contributions of this paper are:

- We present a hardware model to enable CNN inference near the sensor for different vision applications.
- We present a scoring-based approach to reduce computation burden and improve power efficiency for pixel processors.
- A suitable communication structure for pixel data propagation to minimize interconnection.

The remaining sections of this paper are organized as follows. Section 2 discusses the related works in the literature. We discuss the background concept in Section 3. Section 4 provides a detailed explanation of our design. We evaluate our model in Section 5.

## 2. Related Work

There are a number of hardware architectures found in the literature that aim to provide acceleration for CNN applications while reducing computational redundancies [14,15,16,17]. And, there are some approaches that attempt to exploit the high bandwidth available near the sensor interface by bringing the computation closer to the image sensor [7]. In this section, we describe these approaches separately in the same order. Afterward, we discuss and identify the distinction of our proposed model.

While the widespread practice of running a CNN is to use a GPU as the hardware accelerator, their deployment on embedded devices encounters limitations, such as cost and power budget [18,19,20], whereas the computational demand of CNN architecture usually overwhelms conventional sequential processors to meet the real-time performance requirements. This has necessitated the emergence of a plethora of novel architectures. These architectures typically employ large numbers of multiply-accumulate (MAC) units but vary significantly in their interconnection network, memory hierarchy, and dataflow approach. However, most of these works either push hardware performance to the extreme or focus primarily on implementing the computation reduction scheme.

A common approach to designing CNN accelerators considers implementing a generic uniform hardware core for convolution that can be used in a time-shared form to execute different convolution layers. The solution offers flexibility, but the solution’s performance and efficiency vary for different CNN and often suffers from performance and energy degradation. To improve efficiency on CNN inference accelerators, authors in Reference [21] executed convolution operations in CNN as matrix multiplications by rearranging the input feature maps. However, the overhead of memory accesses and execution times associated with the rearrangement is large.

In Reference [22], the authors proposed a high accuracy accelerator for convolutional layers where zero skippings and clock gating are used to minimize computations. Even though the design improves power consumption, it does not fully exploit the redundancies available within the computation model.

In Reference [23], the authors introduced a dynamic *precision* and voltage scaling approach for different layers of AlexNet to save power. The work in Reference [24] proposed a power-aware architecture for VGG-based CNNs. These works only concentrate on the efficiency of the hardware and do not discuss in detail how to adopt these schemes on high throughput architectures.

References [25,26] proposed a low-power computation approach based on two types of convolutions for convolutional and pooling layers of the CNN. The lightweight low-precision convolutions predict the max-pooling layer’s output while a high-precision convolution prepares the final result. However, the work solely focuses on reducing the architecture’s power consumption and does not discuss other hardware optimizations. Another problem with the solution is that it always assumes that the convolution layer is always followed by a pooling layer, which is often not true.

Recently, Xilinx has released Deep Learning Processing Unit (DPU), a configurable computation engine for CNNs [27]. The parallelism that can be achieved in DPU is dependent on the target device and application. It includes a set of instructions and supports a wide range of CNN models. While DPU has reported promising results for different CNN models, the approach does not discuss any optimization schemes adopted in its hybrid computing array to reduce redundancy and save power. Furthermore, the feasibility study of coupling the DPU near the image sensors has not been carried out yet.

In focal-plane processing architectures, the processing circuitry is incorporated near the pixels of the image sensor [10,11,28]. Bringing the computation in the sensor can offer distinct advantages in terms of data reduction and power efficiency. However, these designs perform relatively simple operations, for example extracting temporal contrast in each pixel. There are other designs that perform simple convolutional operations to compute low-level features. Despite offering some performance improvement, the data bandwidth is not fully utilized in these implementations.

Darpa’s ReImagine program aims to demonstrate that a single, reconfigurable ROIC architecture can accommodate multiple modes of imaging operations that may be defined after a chip has been designed [8]. The program seeks ROI-based efficient computation models to enable real-time analysis. Even though preliminary works have shown promising results, the landscape of the high-level computation part is still in progress.

Other works exist that employ an array of pixel processors to perform CNN inference [7,29]. In Reference [7], the authors present a software programmable integrated vision chip that can provide sensor-level SIMD parallel processing capability. The architecture embeds a processor in each of its 256 × 256 pixels and uses a microcontroller to operate the vision chip. However, the proposed method performs different parts of the network computation in serial and often relies on external hardware for additional computation. In Reference [29], the authors proposed a pixel processor array (PPA) vision sensor designed to perform CNN inference close to the sensor. The implementation is application-specific and is highly optimized for digit recognition. Here, the authors focus on techniques that require only a small number of bits per pixel. They used a checkerboard storage mechanism to reduce hardware requirements. The adopted representation somewhat compromises the accuracy of the model. In contrast, we perform region-level optimizations in our processing array to avoid a significant decrease in accuracy.

Our method differs from the works mentioned above by using a hierarchical redundancy reduction model into a high throughput inference architecture. We integrate the CNN computation layer directly near a vision-sensor. Our goal is to adopt possible optimization mechanisms in the inference layer that can rip the full benefit of an event-based sensor. The approaches found in the literature provide solutions at best for generic neural network structures. CNN structures are widely being used in different application domains. We believe further optimization can be achieved by designing hardware architectures specifically for image pixels as input features. The pixel processors proposed in our work are specifically designed to explore redundancies to reduce network traffic, power consumption, and improve computation capabilities. Besides, the programmability of the inference structure makes the same chip convenient for different applications.

## 3. Background

In this section, we briefly discuss some background information related to our in-sensor inference architecture.

### 3.1. Exploring Parallelism in Convolution

Convolutional layers are the central part of CNN. The underlying arithmetic unit is a simple but computationally expensive operation. For an input image frame *I*, the equation representing the convolution operation of CNN is shown below:

(1) $$S_{f,y,z}=\sum_{c=1}^{C}\sum_{i=1}^{m}\sum_{j=1}^{m}I_{c,x+i,y+j}K_{f,c,i,j}.$$

Here, the convolution kernel (*K*) is assumed to have a square size of m×m and takes an input frame of C channels. The convolution layers extract features hierarchically in the form of feature maps by convolving the input feature maps (IFM) with weight kernels. The operation is performed on each pixel of the IFM for every output channel. It is possible to exploit the parallelism available within the convolution operation and map the pixels into an array of processing elements to achieve fast computation. A conventional CNN model contains several convolutional layers connected in series followed by fully connected layers. To perform convolution, In our architecture, we map the input frame on an array of processing elements (PEs), where each processor is specifically designed to perform the operation shown in Equation (1) in parallel and generate output for the next layer. PEs within a region are designed to perform the regional convolution operation and facilitate optimum execution.

### 3.2. Spatiotemporal Redundancies

The vision sensor’s primary job is to sense the field of view and generate a stream of pixels for a backend processor corresponding to the scenic event. The functionality is similar to the biological eye-brain setup. Except, a human eye has around 130 million pixels, with only 1.3 million synaptic connections to the brain. The setup indicates a 1% sparsity. Researchers believe this massive sparsity is critical for power and latency trade space and helps avoid sending repetitive information to the brain. Natural images tend to exhibit a positive correlation within local regions, and only a reduced amount of information is passed onto the higher processing part. Based on the information-theoretic considerations, in the biological vision system, the role of early sensory processing is to reduce redundancy [30]. According to this model, the visual system uses an internal model to predict incoming signals and reduces redundancy by removing the repeated components [31,32].

In our approach, we hypothesize that when we divide the image frame into logical regions, the homogeneous regions usually comprise the bulk of the redundancies. We identify these regions by measuring the data variation. The equation for calculating variance in an image patch is shown in Equation (2).

(2) $$\sigma^{2}=\frac{\sum_{i=1}^{n}{\left(x_{i}-\mu \right)}^{2}}{n}.$$

Here, μ represents the mean value. However, the square operation consumes considerable hardware resources. In contrast, we would like to design an efficient hardware circuit to measure data variation in a distributed manner. Therefore, in this work, we simplify operation by calculating the mean absolute deviation (*MAD*) to identify spatially repetitive regions. The formula for calculating *MAD* is shown in Equation (3).

(3) $$MAD=\frac{\sum_{i=1}^{n}\left|x_{i}-\mu \right|}{n}.$$

**Figure 2:**
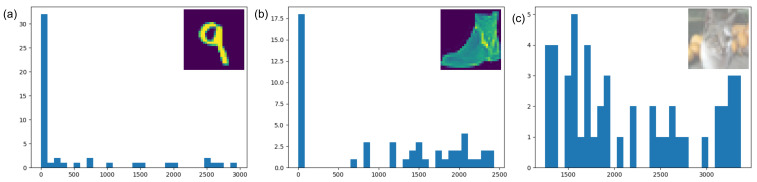
Distribution of mean absolute deviation (*MAD*) where image is divided into 4×4 patches for (**a**) MNIST, (**b**) FashionMNIST, and (**c**) CIFAR10 datasets.

We evaluate our approach by observing the data distribution in MNIST, FashionMNIST, and CIFAR10 datasets. The results are shown in Figure 2. As we can see, for the first two datasets, we have a significant number of regions where the *MAD* value is close to 0. However, for CIFAR10, there is less number of homogeneous regions. This is because, in CIFAR10, we have a very high foreground to background pixel ratio, and the chosen region size is comparable to the actual image size. Still, there are several regions with a *MAD* value of close to zero.

There are 8.3 million pixels in a 4 K image and around 33 million pixels in 8 K. Therefore, it is understood that similar resource management is required for advancing technologies to provide real-time performances. In our approach, we emulate the functionality of the biological vision system to assign computation to regions based on their Spatio-temporal relevance. The model allows us to remove redundancies at different stages of the computation model.

### 3.3. CNN Optimization

CNN optimization is an actively researched area to gain computational efficiency in hardware. Quantization, activation pruning are common practices in CNNs to reduce overheads associated with arithmetic operations. Different encoding based quantization schemes can be found in the literature, such as fixed-point linear quantization, logarithmic quantization, and binarization [33,34,35]. CNN accelerators supporting low *precision* can result in a resource-efficient solution. For example, a single DSP48E2 slice, an available computation unit in Xilinx MPSoC, can perform two 8-bit multiplications simultaneously with a maximum multiplication of 18-bit and 27-bit data [36]. In contrast, a multiplication of two 32-bit data will require four DSP slices. This indicates that the 8-bit model can achieve up to 8 times the computing performance compared to the 32-bit design on an FPGA board with a fixed number of DSP48E2. Therefore, in our approach, we opted for reduced-*precision* operations in low-scoring image regions whenever we can.

## 4. Proposed Model

In this section, we provide a detailed description of our proposed in-sensor CNN inference architecture.

**Figure 3:**
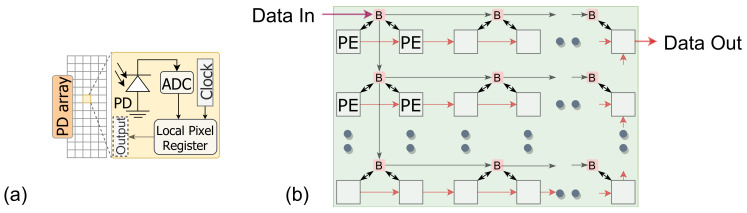
(**a**) Parallel image sensor with analog to digital converter (ADC). (**b**) Data movement within the processing element (PE) array.

The design considers a standard imager depicted in Figure 3a, where each sensing unit in the photodiode array has an analog to digital converter (ADC), and a local memory [37]. The imager data is transferred to a Relevance computation layer (RCL). The design places the image sensor and the computation planes at separate layers. The incoming image frame is logically divided into *M* image regions where N×N pixels reside in each patch. The RCL layer comprises an array of Relevance Computation Units (RCU), where each RCU calculates the relevance score for each region.

The incoming image frames may include pixel regions with noise and interference, which may get misidentified as events. Therefore, before initiating the relevance computation on the image frames, noise-canceling mechanisms can be enforced to eliminate noises and clutters from the pixel data. This step is necessary since natural images are often filled with clutters and noises, which will degrade the performance of event-based processing models. The unwanted noise can be managed by passing the image frame through a low-pass filter or a median filter. For instance, in Reference [38], the authors implemented a weighted median filtering mechanism to filter out impulsive noise with the least area overhead. In Reference [39], the authors present an efficient modified hybrid median filter-based implementation that can improve the visibility of high-quality incoming images with a high frame rate. For this work, a similar technique can be adapted to generate inference-free data while keeping the hardware cost minimal. The noise reduction can be performed as a separate layer before the RCL or integrated within the RCL.

**Figure 4:**
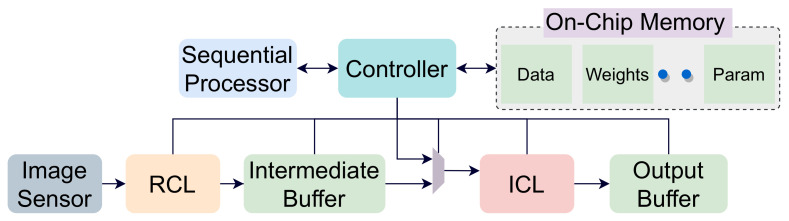
Data flow of our proposed architecture.

**Figure 5:**
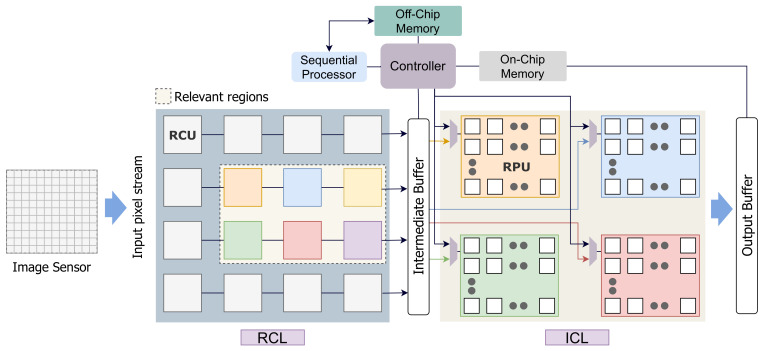
The proposed architecture of our inference computation model. Relevance computation layer (RCL) and inference computation layer (ICL) layers are shown.

The RCL layer is followed by an inference computation layer (ICL) to perform inference near the sensor. In the ICL layer, convolution operations are performed by mapping the image pixels to an array of processing elements (PE). Based on the saliency scores calculated in the RCL layer, relevant regions are scheduled to the ICL for computation. The output of RCL is forwarded to an intermediate buffer for following operations by the ICL. To reduce interconnections, we have one pixel-streaming channel per region between the intermediate buffer (IB) and the ICL layer RPU. These streaming channels can stream pixel values for different image regions in parallel. Besides, there is a global controller unit and an on-chip memory unit to facilitate CNN operations. The controller is used to schedule operations in the computation layers and maintains synchronization. Figure 4 shows the dataflow of our proposed architecture. The off-chip memory is used to store weights and outputs of different layers. This is because a CNN model’s data volume is generally too high to store it locally in on-chip memory. However, we accommodate a global on-chip memory in our design to hide off-chip memory access latency. The controller module pre-fetches the required data from the off-chip memory to the on-chip memory to mask data transportation time. The weights are then forwarded to the PEs, where the computation is performed. The proposed architecture is shown in Figure 5. The intermediate buffer acts as a queue and storage unit for keeping relevant image patches forwarded by the RCU modules. If an incoming image frame is divided into *M* regions, we have *M* RCUs in the RCL layer. However, the number of RPUs is limited in the ICL layer and far less than the number of RCUs. Therefore, image patches are required to be scheduled to RPUs. Here, the intermediate buffer is used to hold image patches while previous patches are processed in the ICL layer. However, it is possible to remove the intermediate buffer from our processing chain. In that case, based on the data demand, the controller will be required to collect image pixels directly from the relevant RCUs and forward them to the RPUs.

In the next subsection, we will provide a detailed explanation of the computation layers and the controller module to illustrate the design flow.

### 4.1. Relevance Computation Layer (RCL)

The RCL layer operates on a region parallel basis. Here, we have a dedicated RCU for each logical region of the incoming image frame. Within each RCU, operations are performed in sequential. The RCU performs the relevance function on each image pixel and accumulates the relevance score for all pixels in a region. For spatial relevance information, we calculate the *MAD* value for each region (shown in Section 3). We use this value to rank the image regions based on predefined thresholds. The regions are categorized into three types: high-relevance (11, regions comprising objects), mid-relevance (01, regions with background context information), and low-relevance (00, homogeneous irrelevant background). The formula to measure spatial relevance score (*SRS*) is shown in Equation (4).

(4) $$SRS=\left\{\begin{array}{c} 11\;,\;\;MAD>Th1\\ 01\;,\;\;Th1\geq MAD>Th2\\ 00\;,\;\;MAD\leq Th2 \end{array}\right..$$

For temporal saliency, we compare the incoming pixel to its temporal neighbors. The number of temporal mismatches within a region is compared against a temporal threshold value to determine temporal relevance (0 or 1). The threshold values used in the RCU are empirical and can be adjusted for different scenarios. A high threshold value will decrease the number of activated ROI regions.

We use 3-bit data to represent each region’s Spatio-temporal relevance, where 2-bits for spatial relevance score (SRS) and a single bit for temporal relevance (TRS). Based on the SRS and TRS values, the computation for a given region is determined. This is shown in Table 1. Here, the TRS value of one accounts for the temporal relevance of that region.

### 4.2. Inference Computation Layer (ICL)

The ICL layer is designed to perform the convolution operations and comprises multiple sub-region that can operate independently from each other. The ICL layer interfaces directly with the RCL layer and operates on the incoming pixels to perform convolution. The generated outputs are stored in an output buffer, from where they are redirected to the memory or the ICL layer to perform the computation for the consecutive convolution layers.

The ICL layer comprises a set of Region Processing Units (RPU) and a group of PEs operating under each RPU. The number of RPUs are less than the RCUs in the RCL plane. Therefore, image regions from the RCL are scheduled on the ICL to perform convolution. While scheduling computation on the RPU layer, the relevance score dictates the nature of computation for image patches. All the RPUs operate in parallel, as well as the PEs within it. However, each RPU receives the pixels associated with that region sequentially. Within the RPU, each PE operates as a pixel forwarding unit to broadcast pixels to all the PEs within the region (shown in Figure 3b). Each PE locally stores its neighboring pixel values required to perform convolution operation and forward other pixels. We used the pixel broadcasting model, indicated in Figure 3b, to fast populate the PEs in different regions. The RPUs receive the global start signal from the controller in parallel. The start signal is then propagated through the PE array, similar to the pixel forwarding network shown in Figure 3b. This way, we can minimize latency by initiating convolution operation on each PE right after it finishes forwarding pixels to the next unit. The output of the ICL is stored in an output buffer (OB). For activation, our design performs ReLU operation. The output data is transferred to the OB in a similar data forwarding network as shown in Figure 3b.

Towards the end of the convolution operations, the output is transferred to a second acceleration module for computing the fully connected layers (or any other layers that cannot be mapped on ICL). Since the computational requirement of the fully connected layer is not immense, it can be implemented in a dedicated hardware, or it can be implemented on a low power sequential processor with vector processing units, such as neon processors [40]. We opted for the latter design option for our implementation, where we stream the output to a low-power processor for further processing.

### 4.3. Processing Element (PE) Design

**Figure 6:**
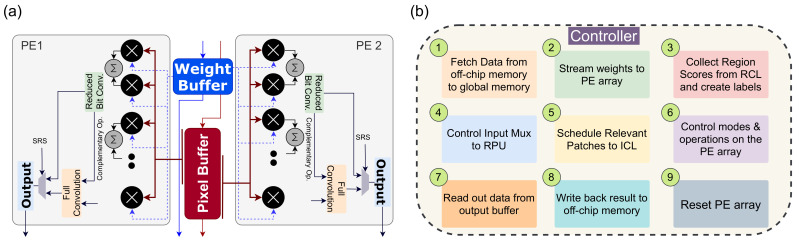
(**a**) Internal design of our PE module. Here, a pair of PPUs share weight buffer and pixel buffer between them. (**b**) Controller functions.

PEs are the building block of our near-sensor architecture. We adopted specific optimization schemes in our PE architecture to minimize redundancy on the input feature map and minimize power consumption. These are specially designed pixel processors that can perform computation on convolution kernel. Each PE has local buffers to store weights, feature maps, and partial output data locally. It reduces access to external memory during the CNN operation. The PE has a vector MAC unit to perform convolution operation. The structure of the PE is shown in Figure 6a.

Each PE performs 8-bits convolution operations on its corresponding pixel. The MAC unit’s 8-bit multiplication module is built from 4-bit multiplication units and executes the Karatsuba multiplication algorithm. All the PEs in a logical region receives a common 1-bit relevance signal from the corresponding RPU. If the relevance bit is 0, PEs of that region only performs an approximate convolution operation based on the most significant bits (4-bits). The full-precision convolution is only performed when the relevance bit is high. Besides, we employ zero-skipping to exploit the sparsity of the weights and data in our architecture. Here, the PE activates a multiplication unit within the vector MAC unit if both the pixel and the weight are non-zero.

#### Resource Sharing

The neighboring PEs in the ICL layers are designed to perform convolution operations on adjacent pixels that have overlaps within their kernel regions. Therefore, it is efficient to share specific resources among them. In our architecture, each pair of PEs along the rows share their weight and pixel buffers. The sharing of kernel weights is straight forward since every PE operates with identical weight values. The pixel regions differ between two row-wise neighbors by one column of pixels for a stride size of 1. Therefore, the pixel values are arranged column-wise in the shared buffer. The sharing of resources in a PE is shown in Figure 6a.

### 4.4. Controller

**Figure 7:**
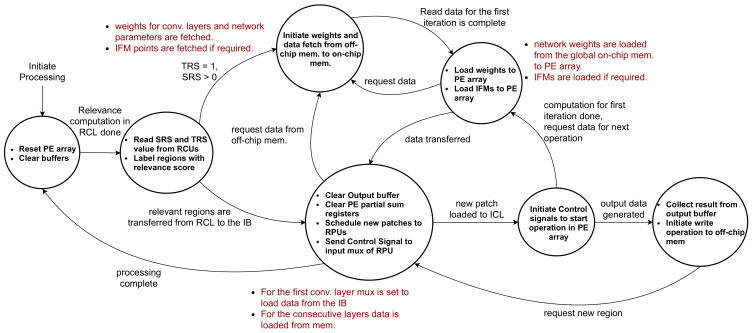
Operation flow of the controller module.

The controller acts as a control unit responsible for maintaining the coherency between different layers of the architecture. The functionality of the controller module is listed in Figure 6b. The unit acts as a custom scheduler that keeps track of the output feature maps in the ICL processors and governs data flow from one layer to the next. At the beginning of the inference processing, once the data is acquired from the RCL layer, the controller sends the global start signal to the ICL processors, and the processing begins. The output of the ICL is multiple feature maps correspond to different output channels. The controller knows the time quantum required to generate each of these feature maps and forward them to the output buffer as they are prepared. As mentioned before, an RPU in the ICL layer operates on an image patch at a time. Here, the Controller schedules image patches from the RCL layer to the ICL layers. The controller module handles the accumulation and transferring of data to the intermediate buffer at the right cycle. Here, the CNN model may require multiple iterations on the ICL layer to complete all the convolutional layers’ computation. This is accomplished by move output data and weights among the ICL layer, intermediate buffer, and on-chip/off-chip memory. Here, the controller unit governs communication and maintains synchronization. The controller ensures that the RCL does not forward the next image frame while the ICL is busy computing the previous one. When ICL finishes the convolution operations, the output is forwarded to a sequential processor where fully connected layers are implemented. While sending pixel data of the feature maps to the ICL, the controller ensures routing of overlapping pixels for convolution. Besides, the controller can update the weights stored in the ICL layer PEs. Figure 7 illustrates the operation flow of the controller module. The controller initiates processing by resetting the PE array in the ICL layer and clear all buffers. Then, it reads the saliency data and labels relevant regions to indicate the type of computation. If no new relevant regions are found, the controller goes back to the initial state. Otherwise, necessary data and parameters are read from the off-chip memory to the global memory and then forwarded to the RPUs. The data can be pre-fetched to hide the latency of off-chip memory reads. Once weights are loaded, image patches are scheduled to the RPU, and a start signal is sent to begin computation on the PE array. The controller sets up the control signal for the input mux of the RPUs to ensure appropriate IFMs are loaded to the RPU. The controller ensures a continuous stream of weights and IFM data at regular intervals to the PE module to ensure convolution operation. Once the output is generated and forwarded to the output buffer, the controller transfers the data to the global on-chip memory. From there, the data is written back to the off-chip memory if required. At this point, the controller clears the output buffer and PE registers to begin computation on the next image patch. Once all computation for an incoming image frame is done, the controller moves back to the initial state.

### 4.5. CNN Layer Scheduling and Execution

The architecture handles CNN models with multiple layers by scheduling them in the ICL layer. For the first convolution layer, input feature maps are forwarded from the RCL layer to the ICL layer. The RCL propagates the SRS and TRS score to the controller module. For regions with TRS value 0, we do not carry out the convolution, and outputs generated from the previous frame is reused. The controller does not map these regions to the ICL. This is the same case for regions with an SRS score of 0. Therefore, the controller only forwards the relevant regions with a non-zero SRS and TRS score from the RCL layer to the ICL. At the beginning of the execution, the RPU fetches the necessary data to populate the PEs with appropriate weights. Then, the IFMs are loaded onto the PEs in the ICL layer as described before. Within each PE, the convolution operations are executed in parallel. To initiate execution in the PE, a start signal is sent from the RPU to all the PEs. Besides, one single bit relevance signal is sent to dictate the nature of the computation (4-bit or 8-bit). The ICL layer’s output is forwarded to the output buffer (OB), from where they are routed to the appropriate memory for further computation. While routing the data from the OB, max-pooling operations are performed if required by the model. For the subsequent convolution layers, IFMs are rerouted from the output feature maps of the preceding layers to the ICL layer. Here, we use a multiplexer to mux data between the IB and the global on-chip memory, as shown in Figure 5. The controller synchronizes this operation by loading new weights to ICL before initiating the next layer operation’s start signal. Once all the convolution layers are computed, the OFMs can be either saved back to the off-chip memory or sent to an accelerator/sequential processor for further processing. The design flow is shown in Figure 4.

### 4.6. Relevance Propagation

**Figure 8:**
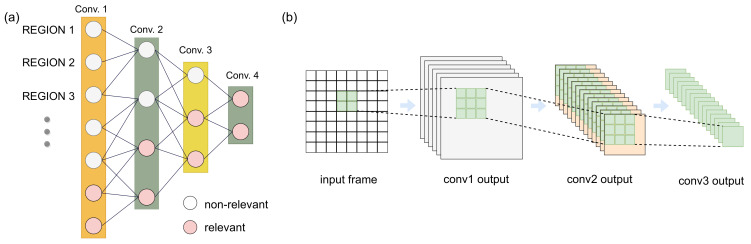
(**a**) Relevance propagation of regions across different convolutional neural networks (CNN) layers. (**b**) Region-level computation initiation.

Usually, CNN models comprises multiple convolution layers where each layer feeding to the next. However, as we have described, we only compute relevance on the incoming image frame from the sensor. As the data propagates through the model, computation of an image region depends on the output of the multiple image regions generated from the previous layer. This is due to the pooling and window operations of the CNN. In an image sensor, a new frame is generated at each time quantum. Generally, consecutive image frames do not have drastic changes in all its pixel regions. Therefore, it is efficient to preclude static region from repeated operations even within the CNN model. In our architecture, we opted for a systematic relevance propagation model to minimize computation on the consecutive CNN layers. Here, we tag each incoming image frame region with the calculated relevance score. As the feature map regions propagates through convolutional layers and merge with each other due to overlaps, we merge the relevance score for those regions, as well. The computation is performed in the controller module and accomplished by a bit-wise OR operation between the relevance scores of the regions. For instance, if there are four OFM regions that are contributing to an IFM region of the following layer, the computation will be skipped if all the OFM regions have TRS of 0. The relevance propagation is explained in Figure 8.

### 4.7. Region-Level Event vs. Pixel-Level Event

As discussed above, in the RCL layer, we identify important events in a region level. This means we label image patches with a relevance score not individual pixels. RCUs are tasked to compute the relevant data in a distributed manner. The approach is in contrast with popular approaches where events are detected on a pixel-basis. For instance, in Reference [41], the authors demonstrated an event camera simulator, where events are detected based on motion, optical flow, depth, and other indexes. In their work, the rendering engine operates on a pixel basis. The problem behind this approach is that if we design a high-level inference engine that collects pixels from this event camera, the inference module will not be able to leverage the events detected by the earlier stages. At best, the inference engine can operate whenever there is a change in events. However, every time there is a change in events, the high-level reasoning algorithm will be performed over the whole frame. With our region-level event detection approach, the designed inference layer can localize the computation, and any new events will initiate computation only in a specific region. This is shown in Figure 8.

## 5. Results

### 5.1. Evaluation Infrastructure

We implement the full RTL-to-GDSII flow on Application Specific Integrated Circuit (ASIC) for the Image Sensor at the block level at 0.9 V supply voltage using the TSMC standard cell library for 90 nm technology. We used Synopsys VCS to simulate and behavioral check of the RTL. These RTLs are written in a hardware description language (HDL). Then, Synopsys Design Compiler converts the RTL to gate-level netlist. Maintaining the Synopsys design constraint, we extract the GDSII for fabrication. Besides, for evaluating and developing the concept, we start the implementation on the Virtex Ultra scale plus FPGA board (xcvu440) using Vivado design suite 2018.2. While developing in the FPGA, we emphasize on reusing the RTL design in ASIC. The RTL is initially analyzed in Vivado to achieve maximum frequency by breaking the critical and high fan-out paths and optimize the RTL to reduce the critical path delay.

### 5.2. Implementation Details

In this work, we implemented our architecture for 8-bit integer operations where PEs are designed to perform convolution. The attention module on the RCU operates independently on each image region and calculates the data variation. For low-relevance regions, PEs are only activated to perform 4-bit convolutions.

In this implementation, we designed each RPU with 8×8 PE units. Here, we designed eight instances of RPU that comprise the ICL. This indicates that the ICL layer will have 512 PEs corresponding to 8 RPUs. These RPU can be programmed to realize one or multiple consecutive CNN layers. The data transfer time between the off-chip memory to the on-chip acts as a performance bottleneck to the architecture. To minimize this, while loading input data from the off-chip memory to on-chip memory, we use a ping-pong buffering scheme to hide latency.

**Figure 9:**
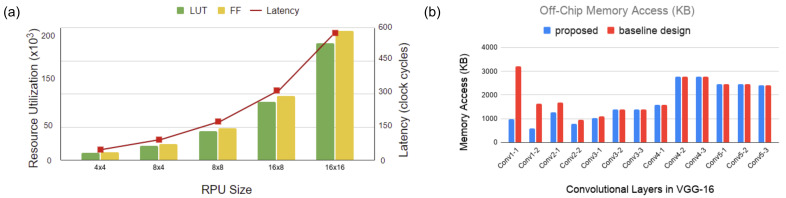
(**a**) Analysis for different RPU region sizes. (**b**) Off-chip memory access analysis for VGG16 model.

We simulate our architecture with different region sizes in the ICL. Figure 9a illustrates our analysis. Here, the y-axis on the right indicates the number of clock cycles for latency in RPUs. The y-axis on the left denotes the number of units utilized for resources, such as flip flops and LUTs. The figure indicates an increase in area overhead and latency with the increase in region size. In contrast, the performance of ROI degrades in a broader region because the regions cannot preclude redundant information. In addition, it asserts high latency that breaks the benefit of parallelism. Based on this analysis, in Figure 9a, we consider the 8×8 region size as the optimal region size in the ICL layer.

### 5.3. Performance Analysis

Each RPU in ICL has 64-PEs, and each RCU in RCL operates on 64 pixels. The resource utilization in the FPGA and layout extracted parameters in ASIC have been tabulated in Table 2 and Table 3. We report a maximum frequency of 350 MHz and 380 MHz in FPGA and ASIC, respectively. We provide the mentioned frequency to every module in our design to maintain synchronization. From Table 3, we can see that on average, each PE has an area overhead of 145,00 µm${}^{2}$. The survey in Reference [42] reports that the minimum silicon footprint of an ADC in 90 nm technology is 59,000 µm${}^{2}$. Therefore, to avoid performance degradation, we found that using an additional computational layer in parallel to the sensor is the most viable option for sensor level CNN integration. However, the total area will shrink considerably with improved silicon technology. In our implementation, we have a dedicated RCU for each input image region and eight RPUs in the ICL layer for inference computation. Therefore, for the VGG16 model, with the input frame size of 224×224, we will require 784 RCUs in the RCL layer. However, RCUs are lightweight modules and consume limited resources. The 784 RCU will account for 42 k LUTs and 22 flip-flops, comparable to a single RPU. For this design, the 8 RPUs in the ICL layer will require 350 k LUTs and 390 k flip-flops in total.

**Figure 10:**
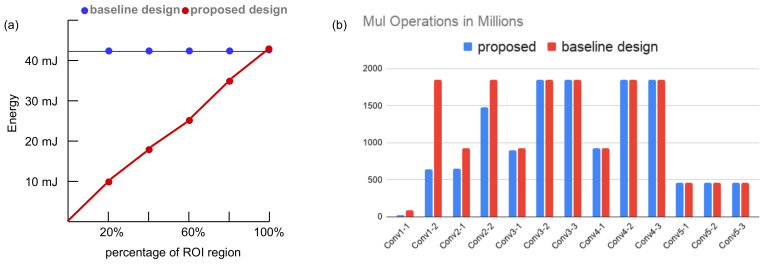
(**a**) Energy consumption analysis of the convolution layer in our proposed architecture. (**b**) Computational redundancy analysis for VGG16 model.

We exhibit the benefit of integrating the relevance computation layer by analyzing the energy consumption of the RCU and RPU in ICL. The energy overhead in our architecture is the added RCUs in the RCL layer, and the RCUs are active at all times. However, Table 2 and Table 3 indicate that, compared to an RPU, an RCU only consumes 0.5% of energy, whereas an inactive RPU only draws leakage power, and the leakage power in our design is 2.5%∼3.0%. The RCL and ICL layers are designed to compute the convolution operations of the CNN model. Considering our design to a design without the RCL layer, we found that our architecture will save energy in convolution operations, if the ROI is less than the size of the image frame. We tested it on the input image stream of 224×224 frames to a VGG16 model and computed the energy savings on the first convolutional layer. We see that the energy consumption decreases in our proposed architecture as the size of the ROI decreases, whereas a baseline architecture (design without saliency computation layer) consumes constant energy for convolution layers regardless of the size of the ROI. This is shown in Figure 10a. The sequential processor in the architecture is used to implement other computational layers in the high-level processing model. For an arm A53 core implemented as the sequential processor, the estimated power consumption is 500 mW.

The proposed architecture eliminates computational redundancies by reducing the number of multiplication operations in CNN layers. We analyzed the reduction in computational redundancies on the VGG16 model. This is shown in Figure 10b. Here, we assume that the size of the ROI in the incoming image frame is 30% (estimated from the first three entries in Table 4). The figure indicates that the proposed model performs fewer multiplications than a baseline design (without visual attention) in the first five layers. For the consecutive layers, the ROI size increases due to the relevance propagation model and the computation load is equal for both designs. The computational savings in the first five layers result in around 13% reduction in total energy consumption. Next, we investigate the off-chip memory access for the ICL layer. The off-chip memory is accessed to fetch weights of the convolution layers and store OFMs containing only the relevant regions. The OFMs may need to be read back to the on-chip global memory pool for the next layer operation if the global memory is not large enough to accommodate OFMs. It is the same if the CNN model has intermediate functions that need to be performed on a separate module. Figure 9b shows the off-chip memory access for different convolution layers of the VGG16 model. Here, we compared our proposed design with the same baseline design as described above. We see a considerable reduction in memory access on the first few layers (up to 3.3× decrease on the first convolution layer). However, in our calculation, we do not consider any compression mechanism. Further reduction can be achieved by adopting a suitable compression mechanism in the architecture. For instance, in Reference [22], the authors employed a Run-length compression (RLC) mechanism to reduce the off-chip memory accesses. The RLC coding exploits the zeros in the feature maps to save off-chip memory bandwidth. A similar encoding approach can be explored in our design to curtail memory read/write volume.

**Figure 11:**
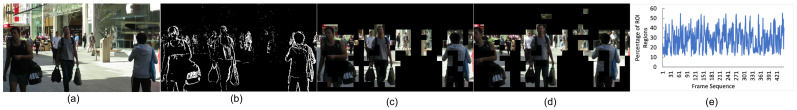
Region-level temporal relevance computation. (**a**) Original image, (**b**) computed motion pixels, (**c**,**d**) identified relevant image regions at time t and t + 1, (**e**) Avg percentage of ROI regions over time.

Next, we seek to estimate the typical size of the ROI in an image frame. We explore different object tracking datasets and developed a simulator to simulate the behavior of region-level event detection of our image sensor. We specifically tested on MOT17 dataset for a real-world scenario [43]. The dataset contains different video clips of people walking around various public places (intersection, square, etc.). We use four different MOT17 datasets to evaluate our temporal relevance model. Table 4 indicates the average number of non-relevant regions for each dataset. As we can see, for the first three datasets, on average up to 75% of the regions can be avoided on each frame for repetitive computation. However, for the last dataset, we have a comparatively less number of irrelevant regions. Because, in this case, the images are captured from a moving camera. Therefore, it is understood that our attention-based approach provides greater power and computational savings for static camera positions. Figure 11 provides a pictorial view of our ROI simulator. Besides, we calculated the spatial redundancy on MNIST and FashionMNIST dataset. The average spatial redundant regions are 50% and 29%, respectively. The results indicate that our architecture will save power at all times.

Next, we evaluate the impact of region-aware processing on the performance of the different inference models implemented in our sensor architecture. We found that we do not see a drop in accuracy when we perform region-aware training (replacing non-relevant regions with zero-valued pixels during training). This is tested on two different models (Lenet-5, VGG16) for three different datasets (MNIST, FashionMNIST, and mosquito species [44]). However, the threshold value used to identify non-relevant regions needs to be adjusted for different datasets. Nevertheless, we were able to exclude 30-to-50% regions for all three datasets.

In our architecture, we perform low-precision operations on regions that have lower relevance scores. The approach was inspired by Reference [45], where the authors used different bit-width operations in different CNN layers. We evaluate whether the approach affects the detection accuracy of the implemented inference. We tested it on the YOLOv3 model and calculated the *precision* metric for the MOT17-05 dataset. *Precision* is defined as the ratio of correct detections to the sum of correct detections and false detections. A substantial value of the *precision* metric indicates high detections. The formula is shown in Equation (5).

(5) $$Precision=\frac{TP}{TP+FP}.$$

**Figure 12:**
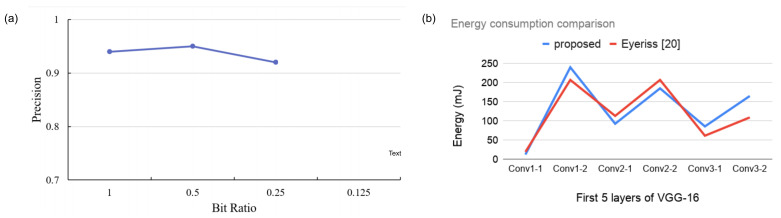
(**a**) *Precision* of our object detection model on MOT17-05 dataset. Here, bit-width ratio indicates the ratio between reduced-precision and full-precision convolution operation. (**b**) Energy consumption comparison with Reference [22] for the first 6 convolution layers of VGG16.

Here, TP indicates True Positive, and FP indicates False Positive. The calculated *precision* data is shown in Figure 12a. The figure suggests that the detector can identify objects on images with reduced bit-width on non-relevant regions.

### 5.4. Performance Comparison

Our architecture’s novelty relies on the implementation of the convolutional layers mapped on an array of pixel processors. We evaluated the performance of our architecture and compared them to a similar work found in the literature. The authors in Reference [46] presented an inference architecture that fully embeds a CNN network on a pixel processor array (PPA) near the vision sensors and demonstrated results for the MNIST dataset. However, the approach does not perform attention-based processing like us. We evaluated our architecture with them to make a fair comparison. The result is shown in Table 5. For the first two convolution layer, our proposed architecture can exhibits 5.1× speed-up compared to Reference [46]. Therefore, we can argue that our architecture outperforms the work in Reference [46]. Next, we compare our work to an FPGA-based SIMD CNN accelerator design [47]. The results are shown in Table 6 which indicated performance improvements in our design.

We performed an energy consumption analysis of our proposed model and compared the work with Reference [22]. The results are shown in Figure 12b. We compared the energy consumption for the first six convolution layers of the VGG16 model. As the figure suggests, our proposed model reports better energy management for the first few layers of the VGG16 model. However, the work in Reference [22] starts to save more energy from the 5th convolution layer and onward. The result is aligned with our previous computations, as our earlier results suggest that the ROI regions spread across the image frame as data propagates through the convolutional layers. Nevertheless, it should be noted that the work reported in Reference [22] exploits zero values in the IFMs to save energy. In our architecture, it is possible to attain greater energy savings by adopting a similar methodology.

The novelty of our work relies on the attention-based CNN processing near the sensor and relevance propagation. Therefore, we focused on highlighting the region-based computation model in this work. However, the modular nature of our design allows the integration of additional optimization methods to improve performance and reduce computational redundancy. For instance, in Reference [48], the authors presented MobileNetV2, which can significantly reduce the memory footprint needed during inference computation. The PE array can be conveniently configured in our architecture to implement depthwise separable convolution layers to carry out CNN operations. There are other optimization schemes that can be integrated within our attention-based approach [49]. In our future work, we plan to explore methods to combine optimization mechanisms to improve inference performance in the sensor.

## 6. Conclusions

This work presents an in-sensor inference processing architecture for reducing redundancies by executing only the relevant regions. We leverage the insights of biological vision systems to reduce Spatio-temporal redundancies in an image sensor. The proposed method includes a novel relevance score-based computation approach that facilitates fast execution of CNN operations directly near the image sensor. We provide efficient data management and communication mechanism to facilitate efficient operation. A rule-driven controller module is proposed that can be configured to facilitate similar CNN models using the same architecture. In our future work, we plan to investigate data-driven approaches to perform inference operations in the sensor. The simulation result of our architecture shows that considerable power saving is possible in redundant regions with a significant speedup.

## Figures and Tables

**Table 1:** Inference computation based on the salience score.

TRS	SRS	Region Processor	Region Output
1	11	**Active**	Driven by current state (full-precision)
1	01	**Active**	Driven by current state (reduced-precision)
0	(11/01)	Inactive	Driven by previous state
(0/1)	00	Inactive	Forced to Zero

**Table 2:** FPGA resource utilization of the RPU in the ICL.

	LUT	FF	DSP	LUTRAM	Frequency (MHz)	Dynamic Power (W)
PE	682	725	2	-	350	0.023
RPU	43,727	48,430	128	-		1.082
RCU	53	27	-	8		0.005

**Table 3:** Layout extracted parameters of RPU and Relevance Computation Units (RCU) module.

-	-	Power (mW)				-	-
Module	Area (m${}^{2}$)	Internal	Switching	Leakage	Total	Delay (µs)	Freq. (MHz)
RPU	925,685	8.67	5.191	0.428	14.289	0.413	380
RCU	1715.2	0.279	0.005	0.008	0.293	0.17	380

**Table 4:** Region-level temporal relevance analysis on MOT17 datasets.

Dataset	Description (30 fps)	Resolution	Avg. ROI Size
MOT17-08	Crowded pedestrian street (stationary camera)	1920 × 1080	41.60%
MOT17-03	Pedestrian street at night (stationary camera)	1920 × 1080	25%
MOT17-01	People in a square (stationary camera)	1920 × 1080	28.29%
MOT17-12	Busy shopping mall (moving camera)	1920 × 1080	69.43%

**Table 5:** Performance comparison I.

-	Convolution Layers Latency	Fmax	Accu.
CNN-PPA (Lenet-5) [46]	160 µs	-	93%
Our Design (Lenet-5)	31.2 µs	350 MHz	98.8%

**Table 6:** Performance comparison II.

-	FPS	Fmax
Accelerator [47] (VGG16)	11.8	150 MHz
Our Design (VGG16)	25.16	350 MHz

## Data Availability

Data sharing not applicable.

## Abbreviations

The following abbreviations are used in this manuscript:

RPU	Region Processing Unit
RCU	Relevance Computation Unit
IFM	Input Feature Map
SRS	Spatial Relevance Score
TRS	Temporal Relevance Score
OFM	Output Feature Map
